# LncRNA MIR155HG Overexpression Promotes Proliferation, Migration, and Chemoresistance in Gastric Cancer Cells

**DOI:** 10.7150/ijms.82216

**Published:** 2023-05-12

**Authors:** Hong Lin, Ruoxuan Ni, Dongdong Li, Mei Zhao, Yan Li, Kexin Li, Qiao Zhang, Changzhi Huang, Shengkai Huang

**Affiliations:** 1 Department of Etiology and Carcinogenesis, State Key Laboratory of Molecular Oncology, Beijing Key Laboratory for Carcinogenesis and Cancer Prevention, National Cancer Center/National Clinical Research Center for Cancer/Cancer Hospital, Chinese Academy of Medical Sciences and Peking Union Medical College, Beijing, 100021, P. R. China.; 2 Department of Clinical Laboratory, National Cancer Center/National Clinical Research Center for Cancer/Cancer Hospital, Chinese Academy of Medical Sciences and Peking Union Medical College, Beijing 100021, P. R. China.

**Keywords:** gastric cancer, MIR155HG, NF-κB, STAT3, chemoresistance

## Abstract

Long non-coding RNAs are thought to play a vital role in a variety of human malignancies. Studies have shown that MIR155 host gene (MIR155HG) acts as an oncogene in several cancers, but the function and its mechanism of MIR155HG in gastric cancer (GC) is still poorly understood. In this study, we determined the biological functions and underlying mechanisms of MIR155HG in GC cells. We found that expression levels of MIR155HG was increased markedly in GC patients' serum. *In vitro* and *in vivo* studies demonstrated that MIR155HG modulated the malignant phenotype of GC cells, such as cell proliferation, colony forming ability, cell migration ability, and tumor growth in nude mice. Next, our results revealed that NF-κB and STAT3 signaling pathways could be involved in regulating the malignant behavior of GC cells. Our rescue experiments showed that inhibiting NF-κB and STAT3 signaling pathways attenuated the phenotypes caused by MIR155HG overexpression. Moreover, cytotoxicity and apoptosis assays revealed overexpression of MIR155HG reduced the apotosis of GC cells induced by cisplatin and 5-FU. Together, our studies suggested that MIR155HG overexpression promoted proliferation, migration, and chemoresistance of GC cells. These results might provide a lncRNA-based target for GC treatment in future.

## Introduction

Gastric cancer (GC) is the most aggressive gastrointestinal carcinoma and is characterized by high rate of metastasis and poor prognosis. GC is the fifth most common cancer and the fourth leading cause of death among cancer patients, there were over 1,000,000 new cases and over 760,000 deaths worldwide in 2020 1. In China, among all cancers, GC-related incidences and mortalities both rank third. In 2016, nearly 396,500 new incidences and 288,500 mortalities were recorded 2. This indicates that GC seriously threatens public health. Growing evidences have illustrated that a large amount of long non-coding RNAs (lncRNAs) were abnormally expressed during GC progression. LncRNAs are RNAs more than 200nt in length with low or no protein-coding capability. A study has shown the involvement of lncRNAs in regulating gene expression at epigenetic, transcriptional, and posttranscriptional levels 3. Numerous studies demonstrated lncRNAs function as an oncogene or tumor suppressor in GC. LncRNAs such as H19, XIST, HOTAIR, MALAT-1, LINC00152, and PVT1, played an important role in GC carcinogenesis 4-9. GAS5, LINC00261, and MEG3 acted as tumor suppressor of GC 10-12.

The MIR155 host gene (MIR155HG), also referred to as the B-cell integration cluster (BIC), is transcribed from a gene on chromosome 21q21 that comprises three exons in total 13. MIR155HG is regarded as a precursor molecule for miR-155 14. Studies have shown high MIR155HG expression levels in lymphomas 15-17, leukemia 18, gliomas 19-21, and non-small-cell lung cancer 22, and MIR155HG functions as an oncogene in these cancers. A study has shown an association between MIR155HG variants rs4143370, rs1893650, and rs11911469 and GC risk in the Chinese Han population 23. Furthermore, compared to normal tissues, an increase in MIR155HG expression was observed in GC tissues 24. However, the function, as well as the mechanism of MIR155HG involvement in GC are still poorly understood.

In this study, our results confirmed the expression level of MIR155HG increased in the serum of GC patients compared to healthy controls (HCs). *In vitro* and *in vivo* studies showed that MIR155HG overexpression promotes growth of GC cells, thereby functioning as an oncogene. Further, MIR155HG played a role in GC cells by activating the STAT3 and NF-κB signaling pathways. In addition, our results also demonstrated that MIR155HG overexpression promotes resistance to cisplatin and 5-FU of GC cells.

## Materials and Methods

### Serum specimens

The serum samples from HCs and patients diagnosed with GC before any medical or surgical treatments were collected at the Cancer Hospital, Chinese Academy of Medical Sciences. The clinicopathological features of patients with GC are shown in Table 1. Tumors were staged using the tumor node metastasis (TNM) grading system. Written informed consent was obtained from each of the participants. This study was conducted according to the Declaration of Helsinki and was approved by the Research Ethics Committee of Cancer Hospital, Chinese Academy of Medical Sciences.

### Cell lines and cell culture

The five human GC cell lines (SGC-7901, MKN45, BGC-823, MGC-803, and AGS), an immortalized gastric epithelial cell line (GES-1), and an immortalized embryonic kidney epithelial cell line (293T) were obtained from Shanghai Institute of Biochemistry and Cell Biology, Chinese Academy of Sciences (Shanghai, China). All cells were cultured in DMEM, or F12 medium (HyClone, USA) supplemented with 10% fetal bovine serum (FBS), 100 mg/ml streptomycin, and 100 U/ml penicillin in a humidified chamber at 5% CO_2_ and 37 °C.

### Extraction and quantitative real-time PCR of RNA

Extraction of total RNA from cultured cells or serum using Trizol or Trizol LS reagent (Thermo Fisher Scientific, USA) following the guidelines specified by manufacturers, and then quantified using a spectrophotometer K5500 (Thermo Fisher Scientific, USA). For RNA reverse transcription, the reverse transcriptase M-MLV kit (#D2629A, Takara, Japan) was used following the manufacturer's instructions. Quantitative real-time polymerase chain reaction (qRT-PCR) was done by employing SYBR Green Premix Ex TagTM II kit (#DRR081A, TaKaRa, Japan) on an ABI 7500 Real-Time PCR instrument (Applied Biosystems, USA). The housekeeping gene GAPDH was selected as internal control to normalize the expression level of MIR155HG. The sequences of qRT-PCR primers used in this study were the following: GAPDH: F: 5'-CCTGGTATGACAACGAATTTG-3', R: 5'-CAGTGAGGGTCTCTCTCTTCC-3'; MIR155HG: F: 5'-GGCTCTAATGGTGGCACAAAC-3', R: 5′-ACAGCATACAGCCTACAGCA-3'; U6: F: 5'-GGAACGATACAGAGAAGATTAGC-3', R: 5'-TGGAACGCTTCACGAATTTGCG-3'; ACTIN: F: 5'-GGGAAATCGTGCGTGACATTAAG-3', R: 5'-TGTGTTGGCGTACAGGTCTTTG-3'; NEAT1: F: 5'-AACGCTTTATTTTCCAGGTGGCA-3', R: 5'-CGGGCTTACCAGATGACCAG -3'.

### Plasmid construction and lentivirus production

The full length of MIR155HG cDNA was synthesized and inserted into the lentiviral pLVX-Puro vector (GENERAY, China). MIR155HG overexpressed lentiviruses were produced by co-transfection pLVX-MIR155HG vector and two packaging vectors psPAX2 and pMD2G into 293T cells using PEI transfection reagent (Roche, Switzerland). Then virus was harvested after transfection at 48 h and 72 h. To create stable MIR155HG overexpression cell lines, cells were treated with puromycin (Sigma-Aldrich Missouri, USA) at 2 µg/ml for 1-2 weeks after 24 h of infection.

### Cells proliferation and colony formation assays

Cell proliferation was performed by employing the CCK-8 kit (#40203ES60, Yeasen, China) according to the protocol detailed by the manufacturer. Briefly, 10 μl CCK-8 was added into every well of a 96-well plate containing 2×10^3^ cells per well at certain points of time (0, 24, 48, 72, and 96 h). Determination of OD value at 450 nm wavelength was using a microplate reader (Thermo Fisher Scientific, USA) following 1 h of incubation. All experiments were carried out in three independent replicates. For colony formation experiments, 500 cells were grown in a 6-well plate and kept in complete medium. After incubation for 14 days, cell colonies were preserved using methanol for 15 min, and then stained with crystal violet (0.1%) for 15 min. The colonies were then counted. All wells were evaluated thrice.

### Cell migration assay

Cell migration assay was performed using Corning 8 mm pore size chamber in a 24-well plate. The upper chamber was added 200 μl serum-free medium suspended with 5×10^4^ cells, and the lower chamber was added 600 μl complete medium. The cells that had managed to migrate to the membranes' bottom surface were preserved using methanol for 15 min followed by staining using crystal violet (0.1%) for 20 min following an incubation period of 18 h. The migration cells were imaged and counted.

### Western blot assay

Cells protein was extracted using lysis buffer (pH 7.4) composed of Tris-HCl (50 mM), NaCl (150 mM), SDS (0.1%), NP-40 (1.5%), PMSF (50 g/ml) and a proteinase inhibitor cocktail (Roche, Switzerland). Proteins were separated on SDS-PAGE, transferred onto PVDF membranes, and blocked using 5% BSA at room temperature for 1 h. Then membranes were incubated with 1:1000 primary antibodies at 4 °C overnight and rinsed with TBST, followed by incubation with 1:3000 secondary antibody at room temperature for 1h. Membranes were washed with TBST thrice, and the specific antigen-antibody complex was detected using a chemiluminescence detection system. All primary antibodies included GAPDH (#5174T), p65 (#8242T), p-p65 (#3033T), STAT3 (#4904T), p-STAT3 (#9145T), PARP (#9532T), cleaved-PARP (#5625T), and cleaved-caspase 7 (#8438T) and secondary antibody HRP-conjugated goat anti-rabbit IgG (#7074S) were purchased from Cell Signaling Technology (CST, USA). Quantitative analysis of protein expression was using ImageJ software with GAPDH as an internal control.

### Cell nucleus/cytoplasm fraction isolation

Cells were digested using trypsin, washed with pre-chilled PBS twice, and lysed on ice for 5 min using lysis buffer containing 10 Mm Tris-HCl (pH 8.5), 140 mM NaCl, 0.5% NP-40, and 1.5 mM MgCl_2_. Cell lysate was centrifuged at 3000 g for 5 min, then the cytoplasmic fraction (supernate) and nuclear fraction (residual pellet) were obtained.

### RNA-FISH

Fluorescent *in situ* hybridization (FISH) kit (#C10910, RiboBio, China) was used for accomplishing RNA-FISH in accordance with the instructions provided by the manufacturer. Cells were harvested when the density on the glass slide was between 60% and 70%. After being fixed, cells were hybridized with specific probes of U6, 18S, and MIR155HG at 37 °C overnight followed by rinsing with saline-sodium citrate (SSC). The nucleus was stained with DAPI. The cells were observed and photomicrographed by a laser confocal microscope.

### *In vivo* tumorigenesis assay

The nude mice used in this study were purchased from Vital River Laboratory (VRL). To create xenograft tumors, 5-week-old female BALB/c nude mice were given a subcutaneous injection comprising 1×10^7^ SGC-7901 cells with empty vector or MIR155HG overexpression. Then the tumor volume (cm^3^) was assessed every third day. Twenty days later, mice were killed and the xenografts were withdrawn and weighed. All methods were performed in the light of the ARRIVE guidelines 2.0 and Declaration of helsinki, and all animal procedures were approved by the Institutional Animal Care and Use Committee of Cancer Hospital, Chinese Academy of Medical Sciences.

### Immunohistochemical staining

Immunohistochemical (IHC) was performed on xenograft tumors fixed in paraffin (Sigma-Aldrich Missouri, USA). Tissue sections were dewaxed with xylene and sequentially hydrated using gradient ethanol after being heated at 60 °C for 2 h. 3% Hydrogen peroxide (H_2_O_2_) was used to inhibite endogenous peroxidase activity, and serum was used to block non-specific sites. Then tissue sections were incubated with 1: 500 Ki-67 antibody (#ab92742, Abcam) at 4 °C overnight, washed, and incubated with homologous secondary antibody at room temperature for 2 h. The tissues sections were stained using hematoxylin and 3, 3-diaminobenzidine solution.

### Cytotoxicity assay

The cytotoxicity of cisplatin (Qilu Pharm, China) and 5-FU (Sigma-Aldrich Missouri, USA) was detected using the CCK-8 assay. 6×10^4^/well cells were plated in 96-well plates with 100 μl complete culture medium. After cultured for 24 h, cisplatin or 5-FU agents with various concentrations were added to the culture wells. 10 μl CCK-8 was introduced and cultured for 1 h after another 24-hour incubation period. Determination of OD value at 450 nm wavelength was using a microplate reader.

### Cell apoptosis assay

The apoptosis detection kit (#AP107, MULTI SCIENCES, China) was used to detect cells apoptosis according to the protocol detailed by the manufacturer. Cells were treated with cisplatin or 5-FU for 24 h were digested, collected and washed. After cells were double-labeled with FITC Annexin V and PI at room temperature for 5 min, flow cytometry was employed to assess cell apoptosis using a flow cytometer (Beckman Coulter, USA) according to the protocol detailed by the manufacturer.

### Statistical analysis

GraphPad Prism version 6 was used for statistically analyzing all data in this study. The data were presented in the form of a mean value with a standard deviation (SD). When a substantial difference existed between groups, the Student's t-test was used. A two-tailed test with a *P*-value of 0.05 was considered significant.

## Results

### MIR155HG is upregulated in tissue and serum of GC patients and is mainly localized in the nucleus

**Figure 1:**
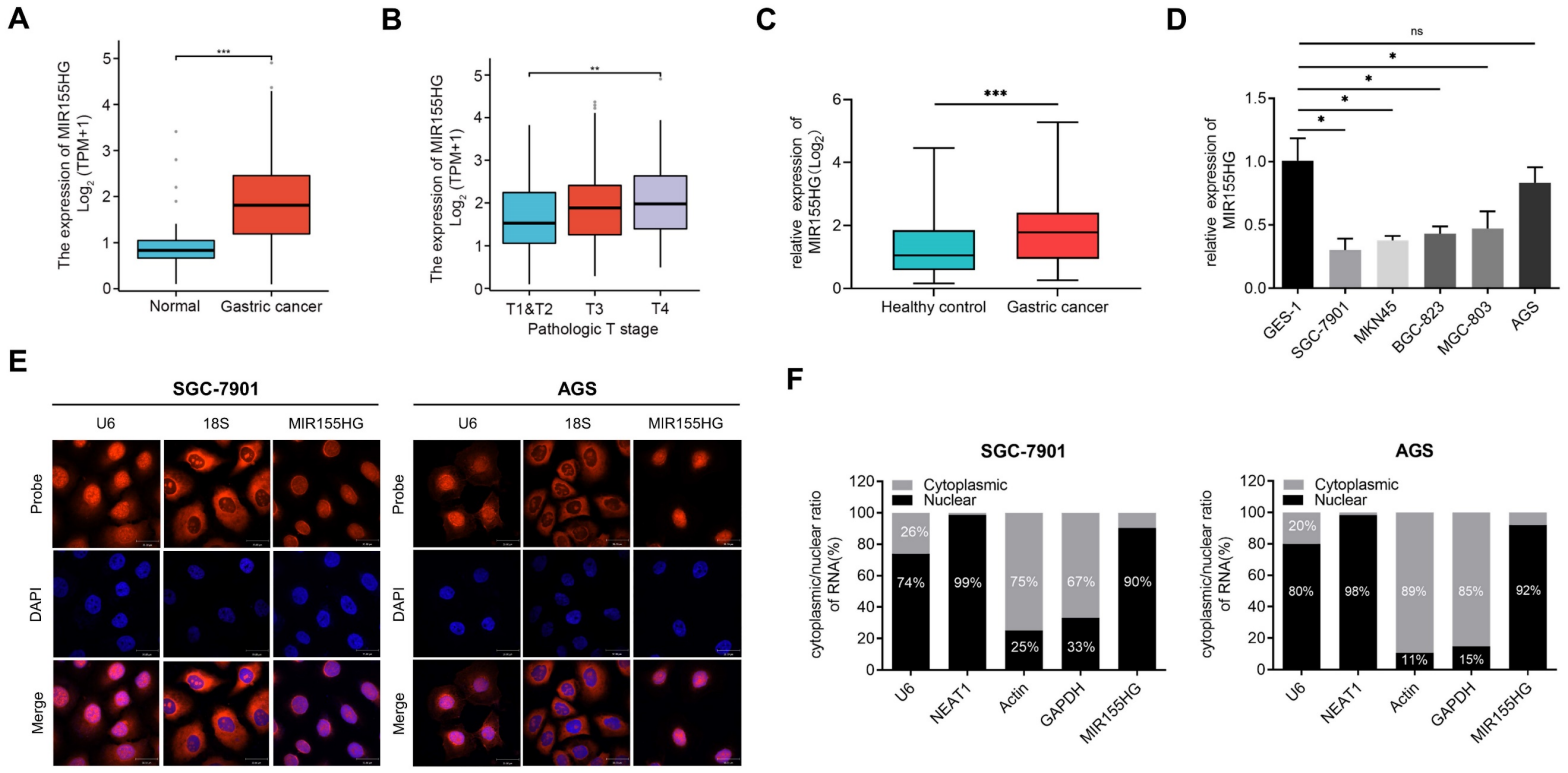
MIR155HG is upregulated in the tissue and serum of GC patients and is mainly localized in the nucleus. **(A)** Increased MIR155HG expression level in GC tissues compared to normal tissues. The information was gathered from the TCGA and GTEx database. **(B)** MIR155HG expression in different pathological stages from the TCGA and GTEx database. **(C)** Upregulation in MIR155HG expression in the serum of GC patients compared to HCs (HC: n = 101, GC: n = 102). **(D)** Relative expression of MIR155HG in 5 human GC cell lines (SGC-7901, MKN45, BGC-823, MGC-803, and AGS) and the human gastric epithelial cell line GES-1. **(E)** The cellular localization of MIR155HG in SGC-7901 and AGS cells was detected using RNA-FISH. The U6 and 18S probes were used as cytoplasmic and nuclear localization markers, respectively. The the cell nucleus was identified by DAPI labeling. Scale bar = 30 μm. **(F)** The distribution of MIR155HG in SGC-7901 and AGS cells was determined using nuclear/cytoplasmic fraction isolation and qRT-PCR. U6 or NEAT1 and GAPDH or Actin were utilized as cytoplasmic and nuclear markers, respectively. All the values are as mean ± SD.***P < 0.001, **P < 0.01, *P < 0.05, ns: no significance.

We retrieved the data on MIR155HG expression from databases including “The Cancer Genome Atlas (TCGA)” and “Genotype-Tissue Expression (GTEx)”, the results revealed an increase in MIR155HG expression level in GC tissues compared to normal tissues (Figure 1A). In addition, the MIR155HG expression was positively correlated with the pathological stage (Figure 1B). Then qRT-PCR was employed for detecting the MIR155HG expression in the serum of 101 GC patients and 102 HC. High MIR155HG expression levels were discovered in the serum of GC patients compared to HC (Figure 1C). However, no correlation was observed between MIR155HG expression and tumor depth, TNM stage, or lymph node metastases in GC patients (Table 1).

Next, we determined MIR155HG expression in five GC cell lines (SGC-7901, MKN45, BGC-823, MGC-803, and AGS) as well as the gastric epithelial cell line GES-1. Compared with GES-1 cells, the expression of MIR155HG in GC cell lines was downregulated (Figure 1D).

Moreover, we performed RNA-FISH to determine the cellular distribution of MIR155HG in SGC-7901 and AGS cells. Our findings showed MIR155HG was mainly localized in the nucleus (Figure 1E). Next, we performed qRT-PCR on RNA extracted from the cytoplasm and nucleus of SGC-7901 and AGS cells. Results also manifested that MIR155HG was primarily located within the nucleus, amounting to 90% and 92% of total MIR155HG in SGC-7901 and AGS cells, respectively (Figure 1F).

### MIR155HG overexpression promotes proliferation and migration in GC cells

**Figure 2:**
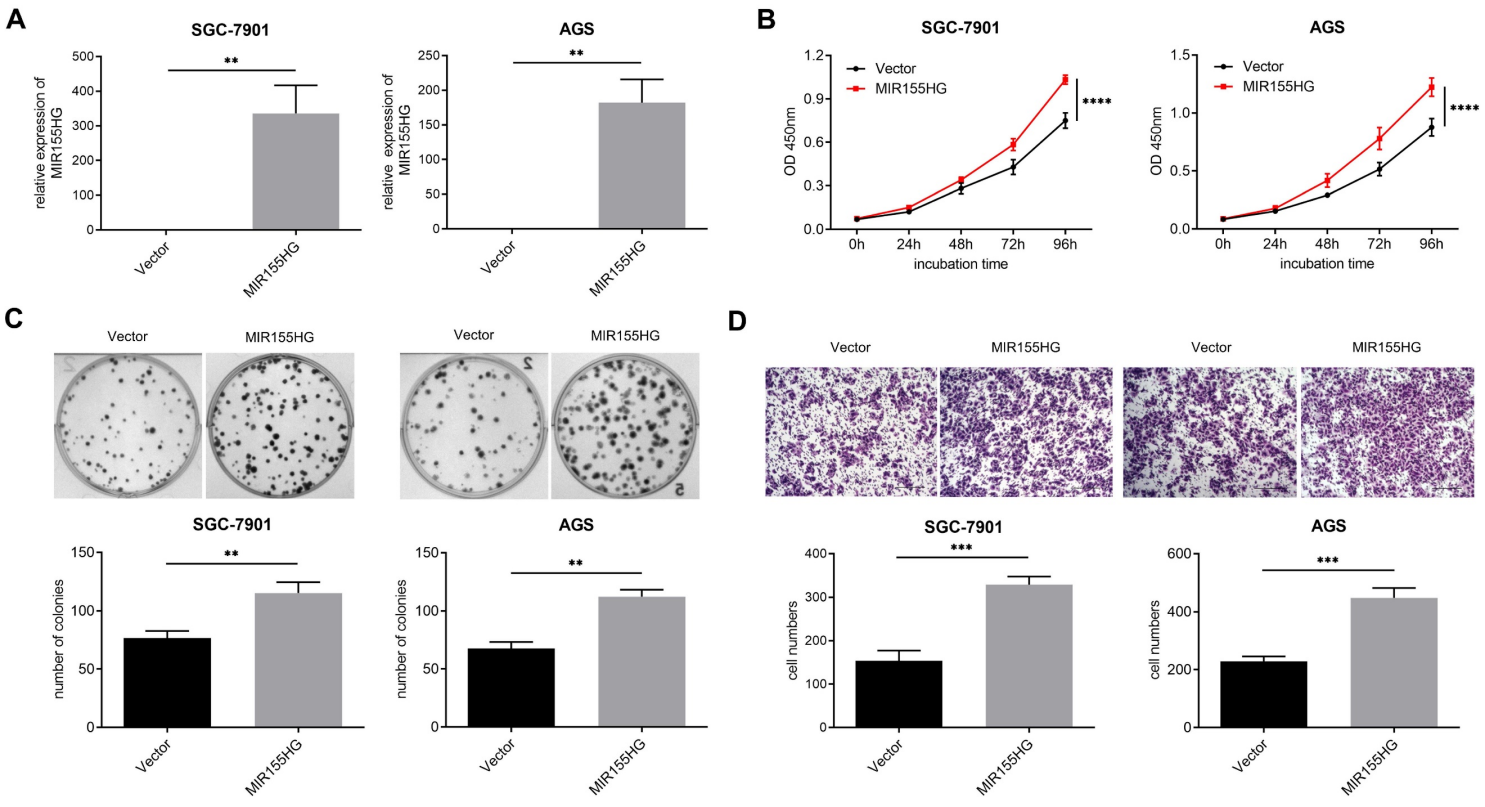
MIR155HG overexpression promotes proliferation and migration in GC cells. **(A)** The efficiency of MIR155HG overexpression in SGC-7901 and AGS cells by qRT-PCR. **(B)** Proliferation assay of SGC-7901 and AGS cells with MIR155HG overexpression by CCK-8 assay. The proliferation experiments were performed three times, each time in triplicate. **(C)** Colony formation assay of SGC-7901 and AGS cells with MIR155HG overexpression. In three tests, colony counts were counted in triplicate.** (D)** Migration assay of SGC-7901 and AGS cells with MIR155HG overexpression by transwell assay. Scale bar = 200 μm. All the values are as mean ± SD. ****P < 0.0001, ***P < 0.001, **P < 0.01.

In order to probe into the biological influence of MIR155HG upon GC cells, MIR155HG overexpressed lentivirus was infected into SGC-7901 and AGS cells, and the efficiency of overexpression was detected by qRT-PCR (Figure 2A). CCK-8 and colony formation assays were performed for assessing the effect of MIR155HG on the growth of GC cells. According to our findings, MIR155HG overexpression facilitated proliferation and colony formation in both SGC-7901 and AGS cells (Figure 2B and C). Then we conducted transwell assay to determine the involvement of MIR155HG in migration of GC cells. MIR155HG overexpression greatly increased the migratory ability of SGC-7901 and AGS cells (Figure 2D) compared to control cells. These results suggest that MIR155HG overexpression affects GC cells proliferation and migration.

### MIR155HG overexpression accelerates tumor growth of GC cells *in vivo*

**Figure 3:**
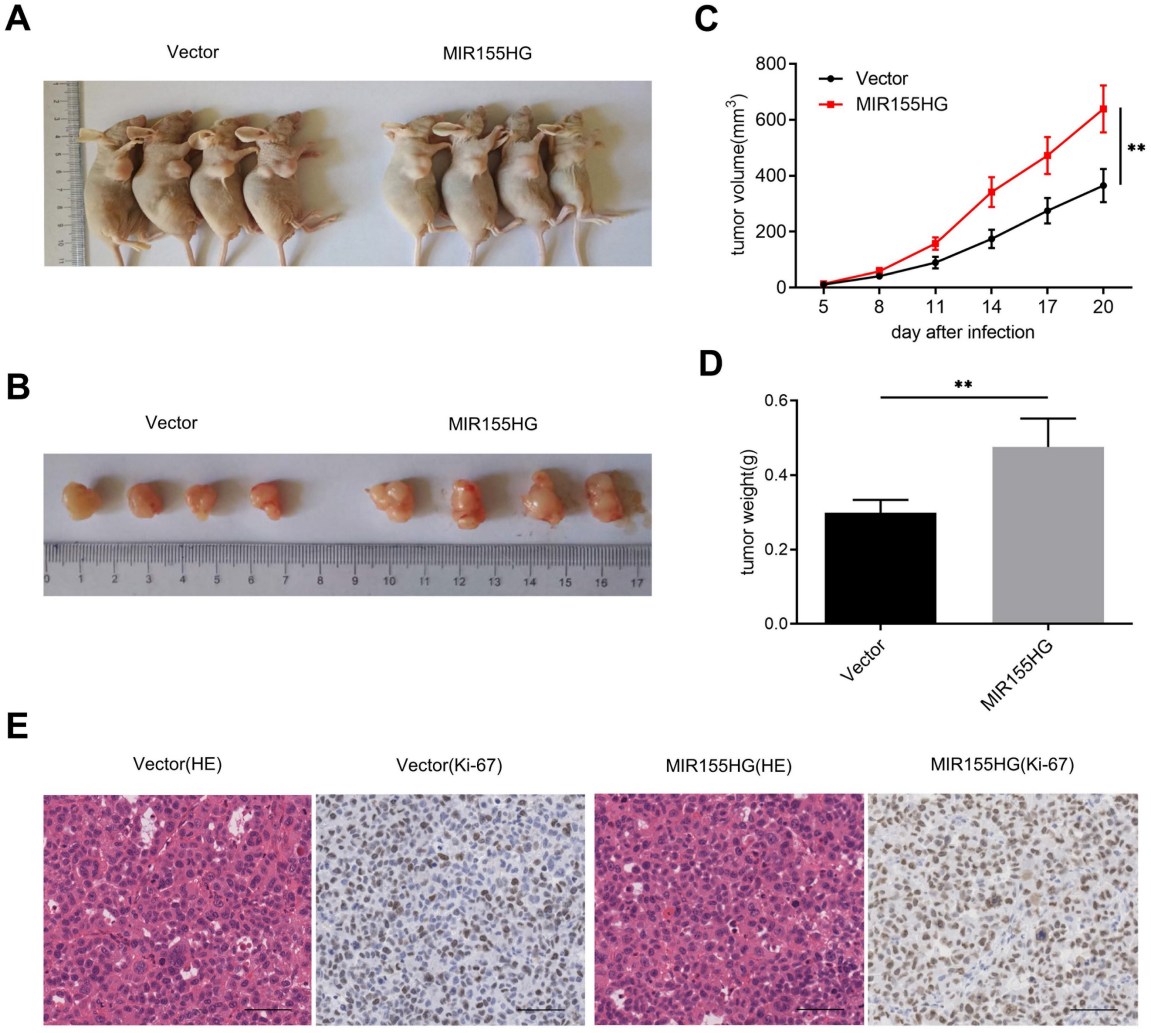
MIR155HG overexpression accelerates tumor growth of GC cells *in vivo*. **(A, B)** Xenograft tumors produced from SGC-7901 cells with MIR155HG overexpression. **(C)** Tumor volumes were measured in each group at the specified time. **(D)** The weights of xenograft tumors in each group. **(E)** Representative photos of HE staining and IHC staining with an antibody against the proliferation marker Ki-67 in each group. Scale bar = 200 μm. All values are as mean ±SD. **P < 0.01.

We established xenograft mice models by subcutaneously injecting SGC-7901 with MIR155HG overexpression or empty vector into nude mice for determining the effect of MIR155HG on carcinogenesis. We observed a significant increase in tumor growth in mice in the MIR155HG overexpressed group than in the control group (Figure 3A and B). And we found MIR155HG overexpression markedly increased the size and weight of the tumor in mice compared to the control group (Figure 3C and D). In addition, IHC results revealed that tumors from the MIR155HG overexpression group showed an increased positive rate of Ki-67 than the control group (Figure 3E). Thus, MIR155HG overexpression promotes tumor growth of GC cells *in vivo*.

### MIR155HG overexpression enhances proliferation and migration through activating NF-κB and STAT3 pathways in GC cells

**Figure 4:**
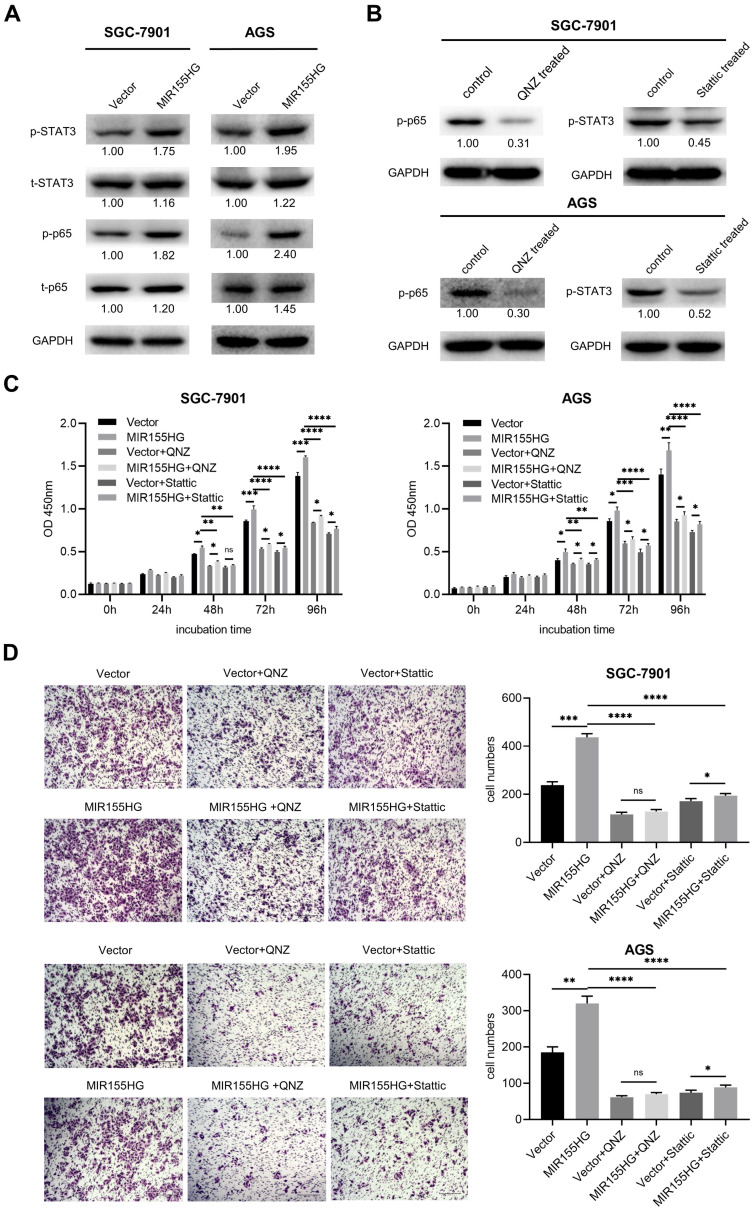
MIR155HG overexpression enhances proliferation and migration through activating NF-κB and STAT3 pathways in GC cells. **(A)** The protein expression of total p65, total STAT3, p-p65, and p-STAT3 in SGC-7901 and AGS cells with MIR155HG overexpression by western blot. **(B)** The inhibiting efficiency of NF-κB inhibitor QNZ and STAT3 inhibitor Stattic in SGC-7901 and AGS cells by western blot. **(C)** Proliferation assay of SGC-7901 and AGS cells with MIR155HG overexpression treated with and without NF-κB inhibitor or STAT3 inhibitor by CCK-8 assay. **(D)** Migration assay of SGC-7901 and AGS cells with MIR155HG overexpression treated with and without NF-κB inhibitor or STAT3 inhibitor by transwell assay. Scale bar = 200 μm. All values are mean ± SD. ****P < 0.0001, ***P < 0.001, **P < 0.01, *P < 0.05, ns: no significance.

Western blot was used for detecting relevant signaling pathways in order to determine the underlying mechanistic process through which MIR155HG promotes GC. NF-κB and STAT3 signaling pathways were reported to contribute to tumor growth, survival, angiogenesis, and metastasis 25, 26. Therefore, we measured total STAT3 and NF-κB p65 and their phosphorylated protein levels in MIR155HG overexpression cells. Western blot results showed a significant increase in p-p65 and p-STAT3 expression levels in SGC-7901 and AGS cells with MIR155HG overexpression (Figure 4A). This demonstrated that MIR155HG could activate the NF-κB and STAT3 signaling pathways in GC cells. Next, in order to determine whether MIR155HG-induced biological effects required NF-κB or STAT3 activation, NF-κB inhibitor QNZ and the STAT3 inhibitor Stattic were employed to treat cells. The inhibition effect of these inhibitors was evaluated by western blot (Figure 4B). The results revealed that blocking the NF-κB or STAT3 signaling pathways could attenuate the proliferative and migratory ability of the cells facilitated by MIR155HG (Figure 4C and D). According to these findings, MIR155HG accelerates tumorigenesis by activating the NF-κB and STAT3 signaling pathways.

### MIR155HG overexpression increases the chemoresistance in GC cells

**Figure 5:**
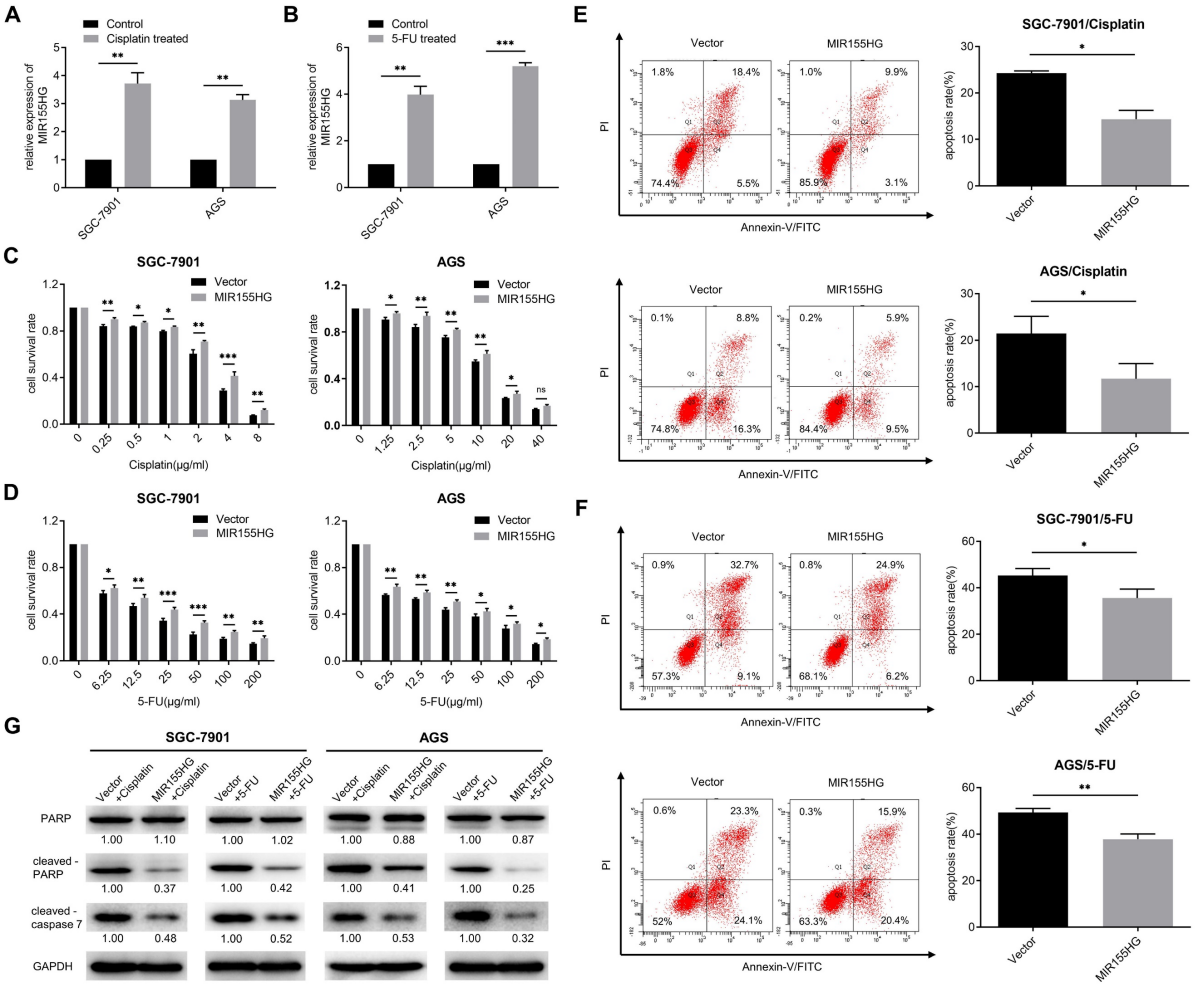
MIR155HG overexpression increases the chemoresistance in GC cells. **(A, B)** The RNA level of MIR155HG in SGC-7901 and AGS cells after cisplatin (A) or 5-FU (B) treatment for 24 h by qRT-PCR. **(C, D)** Cytotoxicity assay of SGC-7901 and AGS cells with MIR155HG overexpression after cisplatin (C) or 5-FU (D) treatment for 24 h by CCK-8 assay. **(E, F)** Apoptosis assay of SGC-7901 and AGS cells with MIR155HG overexpression after cisplatin (E) or 5-FU (F) treatment for 24 h by flow cytometry.** (G)** The protein expression of cleaved-PARP and cleaved-caspase 7 in SGC-7901 and AGS cells with MIR155HG overexpression after cisplatin or 5-FU treatment for 24 h by western blot. All values are mean ± SD. ***P < 0.001, **P < 0.01, *P < 0.05, ns: no significance.

Studies have shown the involvement of some lncRNAs in chemoresistance 27. Hence we determined if MIR155HG was associated with chemoresistance in GC cells. First, we detected the RNA level of MIR155HG in cisplatin-resistant and 5-FU-resistant GC cells. We discovered that MIR155HG expression was remarkably upregulated in both SGC-7901 and AGS cells following 24 h of cisplatin or 5-FU treatment, with MIR155HG expression increasing more than threefold (Figure 5A and B). These results revealed the correlation between MIR155HG expression and resistance to cisplatin and 5-FU in GC cells. The effect of MIR155HG on cisplatin- or 5-FU-induced cytotoxicity in SGC-7901 and AGS cells was then investigated. SGC-7901 and AGS cells overexpressed with MIR155HG were treated with cisplatin or 5-FU for 24 h, and the cytotoxic effects of these drugs on cells were analyzed using the CCK-8 assay. According to the results, a notably higher survival rate was observed in SGC-7901 and AGS cells with MIR155HG overexpression compared to the cells in control group (Figure 5C and D). Further, we determined the effect of MIR155HG on cell apoptosis. Flow cytometry results showed an obviously lower apoptosis rate in SGC-7901 and AGS cells with MIR155HG overexpression than cells in control group (Figure 5E and F). Additionally, western blot results revealed the cleaved-caspase 7 and cleaved-PARP expression levels were substantially reduced in the MIR155HG overexpressed cells (Figure 5G). Taken together, our results show that MIR155HG may induce the chemoresistance of GC cells through inhibiting cell apoptosis.

## Discussion

Studies revealed many lncRNAs are abnormally expressed in GC and and could be used as therapeutic targets 28. lncRNA GAS5 was downregulated in GC and GAS5/E2F1/miR-34c could be the target for anticancer drug development of GC 29. From the database, an increase in MIR155HG expression level was observed in GC tissues. In this study, our results demonstrated an up-regulation in MIR155HG expression level in the serum of GC patients. Furthermore, MIR155HG functioned as an oncogene, encouraging proliferation, migration, and chemoresistance. In addition, MIR155HG promotes GC progression via activating the NF-κB and STAT3 signaling pathways.

NF-κB and STAT3 are two important transcriptional factors participating in oncogenesis 30. The NF-κB signaling pathway is engaged in tumorigenesis via promotion of cell proliferation, survival, epithelial mesenchymal transition (EMT), and angiogenesis 31. Aberrant activation of NF-κB was observed in various human cancers, for instance, thyroid cancer, lung cancer, colon cancer, breast cancer, ovarian cancer and GC 32. NF-κB p65 is an important subunit of the NF-κB signaling pathway. STAT3 is considered as an oncogene and STAT3 activity is dysregulated during tumorigenesis. A study has shown persistent aberrant STAT3 activation in several hematological and solid cancers, including GC 33. In this study, our results showed MIR155HG overexpression increased the phosphorylation levels of p65 and STAT3 in SGC-7901 and AGS cells. Furthermore, inhibiting the NF-κB and STAT3 signaling pathways inhibited the proliferation and migration of GC cells induced by the MIR155HG overexpression, thereby implying that MIR155HG regulates tumorigenesis by activating the NF-κB and STAT3 signaling pathways in GC.

Chemoresistance is a significant cause leading to treatment failure as well as cancer recurrence in GC. Cisplatin, one of the first line anticancer chemotherapy agents for GC in clinical practice, may cause cell apoptosis by causing DNA damage through crosslinking. 5-FU, also one of first line chemotherapeutic drugs used for GC, is an atypical cell cycle-specific inhibitor. 5-FU exerts its cytotoxicity mainly by interfering with the DNA synthesis. GC patients often develop resistance to cisplatin or 5-FU during the treatment. Some lncRNAs were involved in mediating chemoresistance in GC. PVT1 was upregulated in cisplatin-resistant GC tissues and PVT1 promoted the multidrug resistance of GC cells via upregulating the expression of MDR1, MRP, mTOR and HIF-1α 34. MALAT1 promoted autophagy associated chemoresistance of GC cells through increasing the expression of ATG12 by sequestering miR-23b-3p 35. In this study, we discovered that the MIR155HG is linked to the enhancement of cisplatin and 5-FU resistance. In cisplatin and 5-FU resistant SGC-7901 and AGS cells, MIR155HG expression was seen to increase. Furthermore, our results revealed that MIR155HG overexpression could significantly increase the survival rate, diminish the percentage of apoptosis as well as decrease the expression of cleaved-PARP and cleaved-caspase 7 in cells treated with cisplatin and 5-FU. The results indicated that MIR155HG enhances the chemoresistance in GC cells via inhibiting apoptosis of cells. Studies illustrated NF-κB and STAT3 signaling pathways are involved in the resistance of anticancer drugs 36, 37. And we comfirmed MIR155HG could activate NF-κB and STAT3 signaling pathways in GC cells in this study. Therefore, MIR155HG promotes chemoresistance probably through activating NF-κB and STAT3 signaling pathways, however, this needs to be studied further in detail.

## Conclusion

This study demonstrated an increase expression of MIR155HG in serum of GC patients. Furthermore, MIR155HG overexpression enhanced proliferative and migratory capacities of SGC-7901 and AGS cells, and induced chemoresistance. MIR155HG functioned as an oncogene by activating the NF-κB and STAT3 signaling pathways. Overall, our results suggest that MIR155HG could be a potential therapeutic target for GC treatment.

## Figures and Tables

**Table 1:** Correlation between MIR155G expression and clinicopathological parameters of gastric cancer patients (n = 101)

Characteristics	N	MIR155HG		*t*-test *P*-value
		High	Low	
**Gender**				**0.6088**
Male	72	50	22	
Female	29	24	5	
**Age**				**0.8479**
≤60	53	39	14	
>60	48	35	13	
**Clinical stage**				**0.2820**
Ⅰ+Ⅱ	36	29	7	
Ⅲ+Ⅳ	65	39	20	
**Tumor depth**				**0.6175**
T1-T2	32	25	7	
T3-T4	69	49	20	
**Lymph node metastasis**				**0.8845**
Negative	35	26	9	
Positive	66	48	18	
**Distant metastasis**				**0.1829**
Absence	85	65	20	
Presence	16	9	7	

## Abbreviations

5-FU: 5-fluorocrail
BSA: bovine serum albumin
CCK-8: cell counting kit-8
DAPI: 4-6-diamidino-2-phenylindole
DMEM: Dulbecco's modified Eagle's medium
HE: hematoxylin and eosin
GAPDH: glyceraldehyde-phosphate dehydrogenase
NEAT1: nuclear enriched abundant transcript 1
NF-κB: nuclear factor-kappaB
PARP: poly-ADP-ribose polymerase
PBS: phosphate-buffered saline
PVDF: polyvinylidene difluoride
SDS-PAGE: sodium dodecyl sulfate polyacrylamide gel electrophoresis
STAT3: signal transducer and activator of transcription 3
TBST: Tris buffered saline Tween
